# Using artificial intelligence to analyze violence against women in São Paulo

**DOI:** 10.11606/s1518-8787.2026060007099

**Published:** 2026-07-24

**Authors:** Paulo Bandiera-Paiva, Andre Massahiro Shimaoka, Antonio Carlos da Silva, Leonardo Martins Araujo, Débora Dupas Gonçalves do Nascimento, Maria Elisabete Salvador, Marcelo Batista Ribeiro, Kelsy Catherina Nema Areco, José Marcio Duarte, Nacime Salomão Mansur, Dulce Aparecida Barbosa

**Affiliations:** I Universidade Federal de São Paulo. Escola Paulista de Medicina. Departamento de Informática em Saúde. São Paulo, SP, Brasil; II Associação Paulista para o Desenvolvimento da Medicina. Hospital São Paulo. São Paulo, SP, Brasil; III Fundação Oswaldo Cruz. Campo Grande, MS, Brasil; IV Universidade Federal de São Paulo. Escola Paulista de Enfermagem. São Paulo, SP, Brasil

**Keywords:** Violence Against Women, Data Mining, Artificial Intelligence, Data Science

## Abstract

**OBJECTIVE:**

To propose the Health Research Standard Process for Artificial Intelligence framework, adapted from the Cross-Industry Standard Process for Data Mining approach, and apply it using time series and artificial intelligence techniques to analyze data from the Sistema de Informação de Agravos de Notificação (SINAN – Notifiable Diseases Information System) on violence against women in São Paulo, exploring sociodemographic, spatial, and predictive dimensions.

**METHODS:**

The methodology was adapted to health research, replacing the original Business Understanding stage with Research Understanding by incorporating essential technical-scientific elements. We analyzed 80,148 reports of violence against women aged between 20 and 59 living in the city of São Paulo between 2013 and 2023. The analyses included descriptive statistics, calculation of prevalence ratios, decomposition and predictive modeling of time series capturing trends and seasonality, and geospatial analysis of notifications.

**RESULTS:**

We observed temporal patterns and sociodemographic characteristics of the victims, with increasing trends in reports of physical, psychological, and sexual violence after 2015 and seasonal behavior. Among the associations, there was a 32% increase in the prevalence of sexual violence when the aggressor was under the influence of alcohol and a 2.47 times greater risk of sexual violence among pregnant women. The geospatial distribution revealed concentrations of notifications in peripheral areas of the municipality, such as the south, east, and central zones. Predictive modeling indicated that the upward trend will persist over the next 24 months, with estimated rates reaching up to 12 cases per 100,000 inhabitants.

**CONCLUSION:**

The applicability of the Health Research Standard Process for Artificial Intelligence was effective as a model for data analysis and the development of predictive algorithms in public health. It was possible to propose a replicable methodological matrix for future research and evidence-based interventions.

## INTRODUCTION

Violence against women is a serious public health and human rights problem in Brazil, reflecting social, economic and political inequalities that increase women’s vulnerability^1^. This multifaceted phenomenon demands interdisciplinary approaches to understanding and tackling it, including the use of advanced technologies such as data analysis using artificial intelligence (AI) to guide public actions and policies.

According to the World Health Organization (WHO, 2023), approximately 30% of women worldwide have suffered physical or sexual violence from an intimate partner at least once in their lives^1^.

In Brazil, the Anuário Brasileiro de Segurança Pública (2023 – Brazilian Public Security Yearbook) revealed an alarming scenario, with 1,437 femicides recorded in 2022, representing an increase of 6.1% over the previous year^2^. The 2023 DataSenado Institute survey showed that 31% of women interviewed in the state of São Paulo reported having suffered some kind of domestic or family violence^3^.

During the Covid-19 pandemic, this scenario has intensified, with a 22% increase in reports through Disque 180 between March and December 2020, showing that crisis situations tend to aggravate women’s vulnerability^2,4^.

From this perspective, the Sistema de Informação de Agravos de Notificação (SINAN – Notifiable Diseases Information System) is a valuable source of epidemiological data in Brazil, recording cases of domestic and sexual violence and other forms of aggression^5,6^. SINAN is mainly fed by the notification and investigation of cases of diseases and conditions included in the national list of notifiable diseases (Consolidation Ordinance No. 4 of September 28, 2017).

In fact, SINAN is an important tool for health planning, setting priorities, and assessing the impact of interventions. However, the complexity and volume of this data present major challenges for its analysis and for transforming this information into actionable knowledge.

The use of data science and AI is still in its infancy in the context of the Unified Health System (SUS), although some initiatives are already underway, such as InfoGripe and InfoDengue, which use SINAN data for epidemiological monitoring^7,8^, as well as tools such as EAI Pelotas^9^ and Laura Digital Emergency Room^10^, tested in isolation in some SUS units to support hospital care and primary health care management. However, these actions are still occasional and often lack structured methodologies to systematically guide all the stages of construction, validation and application of analytical models.

Therefore, this study aims to propose the Health Research Standard Process for Artificial Intelligence (HRSP-AI) framework as a methodological and replicable structure for creating analytical and predictive models in public health; to apply this structure to the analysis of SINAN data on violence against women in São Paulo, exploring different analytical dimensions such as sociodemographic, spatial, temporal, and predictive dimensions.

## METHODS

### Study Design and Period

This is a methodological study, with a quantitative approach, based on secondary data analysis, carried out at the Department of Health Informatics of the Escola Paulista de Medicina of the Universidade Federal de São Paulo in partnership with the Associação Paulista Para o Desenvolvimento da Medicina, from November 2024 to May 2025.

All the data used is public, anonymized and has been treated in accordance with current ethical and regulatory principles, according to the Brazilian Data Protection Law (LGPD – Law No. 13,709/2018) and Resolution No. 510/2016 of the National Health Council. The study was approved by the Research Ethics Committee of the Universidade Federal de São Paulo, under opinion No. 00263/2023.

### Data Source and Study Population

SINAN data was used regarding notifications of violence against women aged between 20 and 59 living in the city of São Paulo between 2013 and 2023. The extraction showed cases of aggression by unspecified means (ICD Y09), excluding records of self-harm, resulting in 80,148 notifications analyzed.

Complementary sources included: data from the Ministry of Health and the Instituto Brasileiro de Geografia e Estatística (IBGE - Brazilian Institute of Geography and Statistics) (population data for women aged 20 to 59 in São Paulo, SP); information from the SUS Informatics Department (ICD and descriptions); the Brazilian Classification of Occupations; the Cadastro Nacional de Estabelecimentos de Saúde (CNES – National Registry of Health Establishments), the address of the establishments; geographical data from the State Data Analysis System and São Paulo City Hall (map of São Paulo, regions and districts, and latitude and longitude of the establishment).

### Study Protocol

Based on SINAN and the other sources mentioned, a database was built with the variables of interest, covering the stages described in this section of the article.

Given the methodological difficulties in analyzing large volumes of public health data, structured data mining methodologies were used, such as the Cross-Industry Standard Process for Data Mining (CRISP-DM)^11^, recognized for offering a systematic approach to extracting knowledge from massive data^12,13^. Although effective in corporate contexts, CRISP-DM was originally designed for the business sector and does not cover fundamental aspects of health research, such as scientific planning and ethical care in the use of data^14,15^. However, its structure can be adapted to incorporate these elements, making it a robust methodological roadmap for the development of AI models applied to public health^16,17^.

### Adapting the CRISP-DM for Health Research

**Figure 1:**
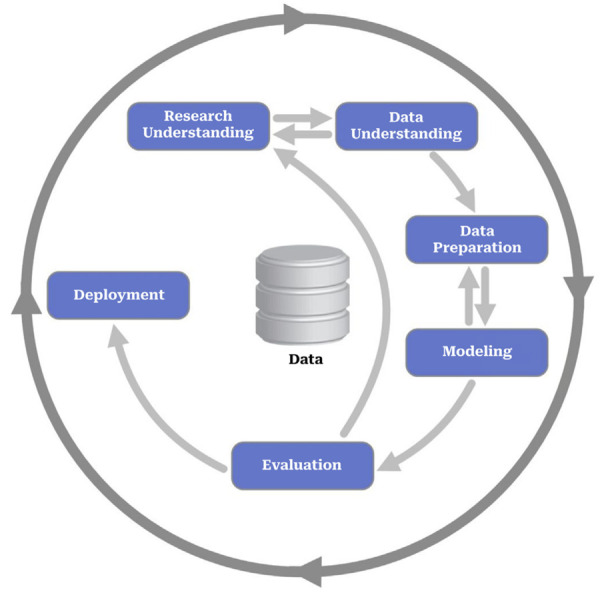
Graphical representation of the Health Research Standard Process for Artificial Intelligence (HRSP-AI).

Source: Adapted from Chapman et al.^7^, 2020.

The traditional CRISP-DM consists of six phases: Business Understanding, Data Understanding, Data Preparation, Modeling, Evaluation and Deployment^7^. To adapt it to the context of health research, the first phase was adapted to Research Understanding, maintaining the cyclical and iterative structure of the original process (Figure 1):

1. Research Understanding: (adapted from the original “Business Understanding”): defines research objectives and questions, elaboration of the justification, literature review, definition of the scope (target population, study period, limitations and coverage), resource planning, data security measures, ethical appropriateness, and methodological definition^18^.
2. Data Understanding: involves initial data collection and exploration, identification of relevant variables, quality and consistency analysis, and preliminary understanding of patterns and relationships.
3. Data Preparation: includes cleaning, transforming, and structuring the data for analysis, including treatment of missing data, normalization, categorization, and creation of new derived variables.
4. Modeling: covers the selection and application of analytical techniques and algorithms for both descriptive and predictive analysis, including time series decomposition and statistical modeling.
5. Evaluation: assesses the results according to the research objectives, using appropriate metrics and validation by health experts.
6. Deployment: refers to the implementation of the knowledge generated from the analysis, including its translation into applicable products such as technical reports, scientific publications, interactive dashboards, or decision support systems. In the context of the HRSP-AI, this stage is aimed at the structured dissemination of the evidence produced and its practical use in the formulation of public health policies and interventions.

### Data Analysis

Descriptive exploratory analyses were carried out, including absolute and relative frequency distributions of sociodemographic variables and characteristics of violence. Prevalence ratios (PR) with 95% confidence intervals (95%CI) were calculated to investigate associations between risk factors and different types of violence.

For the temporal analysis, time series decomposition was applied using the ETS (Error, Trend, Seasonality) method, to identify trends, seasonal patterns, and residual components^19^. Geospatial analyses were carried out to map the distribution of notifications by administrative district in the municipality of São Paulo, based on official geographic data. All statistical processing was carried out using Python, with the Scipy, Statsmodels, Shapely, Geopandas, Sklearn, and Prophet packages for temporal and geospatial analysis.

Exploratory analysis: frequency and distribution analyses were carried out on sociodemographic variables (age, race/color, education, and occupation), type of violence, relationship with the aggressor and associated factors. PRs were calculated to analyze associations between variables, such as alcohol use by the aggressor and the different types of violence.

Data preparation: before applying the decomposition and prediction techniques, the data was prepared using a pipeline that included formatting the dates, aggregating the notifications by month, processing missing data, and integrating it with population data to calculate the rates per 100,000 women. Missing categorical data was standardized and replaced with “Not informed”.

Temporal analysis: ETS time series decomposition techniques were applied to identify trends and seasonal patterns in notification rates per 100,000 women. The decomposition separated the components of: 1) Trend: general direction of the series over time; 2) Seasonality: cyclical patterns within the period; 3) Residual: random variations not explained by the other components.

Data preparation for predictive modeling: data on notifications of violence were prepared using a pipeline that included date formatting, monthly aggregation of notifications, checking for missing data, integration with population data and calculation of rates per 100,000 women.

Predictive modeling: the Prophet algorithm, based on AI techniques, was used to forecast the time series, taking advantage of its ability to capture non-linear trends and multiple seasonal patterns^20^. Prophet was chosen for modeling and forecasting because of its advantages over traditional methods (ARIMA and Holt-Winters), including flexible modeling of multiple seasonalities, capturing non-linear trends and robustness to outliers^20^. The accuracy metrics of the Prophet model were evaluated using for the mean absolute error (MAE) and root mean square error (RMSE) and time series cross-validation with multiple moving windows, as is usual practice in public health time series modeling.

Geospatial analysis: the geographical distribution of notifications by district in the municipality of São Paulo was mapped, identifying concentrations and associating them with the health establishments that made the notifications.

## RESULTS

The analysis of the 80,148 records of violence against women aged between 20 and 59 in this municipality (2013–2023) is presented in three main axes, aligned with the stages of the HRSP-AI framework: (1) descriptive sociodemographic analysis and characteristics of violence (“Data Understanding” stage); (2) temporal and predictive analysis (“Modeling” and “Evaluation” stages); and (3) geospatial analysis (“Modeling” part, focusing on territorial risk patterns).

### Characterization of Notifications

In this stage of the HRSP-AI framework, corresponding to “Data Understanding”, a descriptive analysis was carried out of the sociodemographic variables and the characteristics of the episodes of violence reported (Table 1). The age distribution showed a higher concentration in the younger age groups, with a higher frequency between 20–29 years (39.6%), especially in cases of physical, psychological, and sexual violence, with a progressive decrease in the older age groups. The average age of the women was 34 years (SD = 10.02; 95%CI: 33.98–34.12).

**Table 1:** Sociodemographic characteristics of victims of violence against women. São Paulo, SP, Brazil, 2025.

Variable	n	%
Age group		
20–29	31,741	39.6
30–39	25,453	31.8
40–49	15,587	19.4
50–59	7,367	9.2
Race/Color		
Brown	32,337	40.5
White	32,102	40.2
Black	9,936	12.4
Unknown	4,566	5.7
Yellow	671	0.8
Indigenous	331	0.4
Not informed	205	0.3
Schooling		
No schooling	2,934	3.7
Elementary school	8,633	10.8
Elementary school II	13,142	16.4
High school	26,444	33.0
Higher education	5,442	6.8
Not known	22,729	28.3
Not informed	824	1.0
Occupations (top 10)		
Unemployed	7,764	9.7
Housewife	6,465	8.1
Ignored	6,234	7.8
Housekeeper	990	1.2
Student	966	1.2
Cleaning lady	855	1.1
Saleswoman	682	0.9
Nurse	636	0.8
Nursing assistant	631	0.8
Cashier operator	524	0.7

Source: Sistema de Informação de Agravos de Notificação, 2013–2023 (Notifiable Diseases Information System).

**Figure 2:**
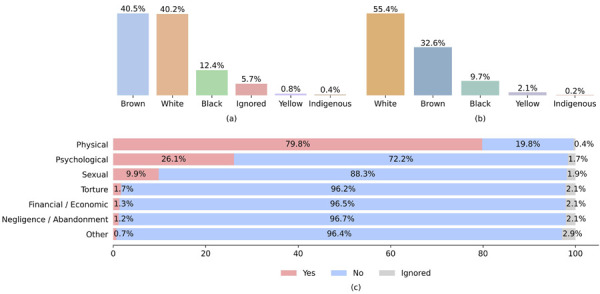
Data on violence against women according to race/color and type of violence, São Paulo, SP, 2025.

As for race/color, there was an over-representation of brown (40.5%) and black (12.4%) women among the victims, considering the city’s population distribution (Figure 2A). Compared to IBGE data (2022), there is a significant disparity, since the general population of São Paulo has approximately 32.6% brown and black people (Figure 2B).

(a) Data on violence against women in terms of seasonality and trends (2013–2023). (b) Forecast of trends and seasonality of violence against women until 2026.

Source: Sistema de Informação de Agravos de Notificação, 2013–2023 (Notifiable Diseases Information System).

In terms of education, 33.0% of the victims had completed high school, while 6.8% had higher education. The high percentage of records with unknown or uninformed education (29.3%) stands out. Occupational analysis revealed the “housewife” category as the second most frequent (8.1%), behind “unemployed” (9.7%).

### Characteristics of Violence

Physical violence was predominant, present in 79.8% of cases, followed by psychological violence (26.1%) and sexual violence (9.8%) (Figure 2C). Regarding the means of aggression, bodily force/beating was the most common (73.8%), followed by threats (18.3%) (Table 2).

**Table 2:** Characteristics of reported violence against women. São Paulo, SP, Brazil, 2013–2023.

Variable	Yes	No	Ignored
	n (%)	n (%)	n (%)
Type of violence			
Physical	63,978 (79.8)	15,855 (19.8)	315 (0.4)
Psychological	20,945 (26.1)	57,863 (72.2)	977 (1.7)
Sexual	7,895 (9.8)	70,757 (88.3)	1,496 (1.9)
Torture	1,344 (1.7)	77,085 (96.2)	1,719 (2.1)
Negligence	934 (1.2)	77,528 (96.7)	1,686 (2.1)
Financial	1,054 (1.3)	77,375 (96.5)	1,719 (2.2)
Other	556 (0.7)	77,255 (96.4)	2,337 (2.9)
Means of aggression			
Bodily force	59,122 (73.8)	19,378 (24.2)	1,648 (2.1)
Threats	14,640 (18.3)	62,224 (77.6)	3,284 (4.1)
Sharp object	4,983 (6.2)	71,733 (89.5)	3,432 (4.3)
Blunt object	4,348 (5.4)	72,277 (90.2)	3,523 (4.4)
Other	4,317 (5.4)	71,117 (90.2)	4,714 (4.4)
Hanging	4,096 (5.1)	72,633 (90.6)	3,416 (4.3)
Firearms	920 (1.1)	75,678 (94.4)	3,550 (4.4)
Hot object	417 (0.5)	76,205 (95.1)	3,526 (4.4)
Poisoning	467 (0.6)	76,079 (94.9)	3,602 (4.5)
Sex of perpetrators			
Male	58,221 (72.6)	–	–
Female	11,527 (14.4)	–	–
Both sexes	6,935 (8.7)	–	–
Not known	3,465 (4.3)	–	–
Motivation			
Unknown	22,361 (27.9)	–	–
Other	20,465 (25.6)	–	–
Not applicable	13,814 (17.3)	–	–
Sexism	13,778 (17.2)	–	–
Generational conflict	7,187 (9.0)	–	–
Street situation	1,062 (1.3)	–	–
Disability	608 (0.8)	–	–
Homophobia, lesbophobia, biphobia, or transphobia	510 (0.6)	–	–
Racism	152 (0.2)	–	–
Religious intolerance	67 (0.1)	–	–
Xenophobia	55 (0.1)	–	–

Source: Sistema de Informação de Agravos de Notificação, 2013–2023 (Notifiable Diseases Information System).

The main perpetrators of violence were males (72.6%), with spouses being the most frequent aggressors (30.2%), followed by ex-spouses (13.3%) and strangers (14.8%) (Table 3).

**Table 3:** Relationship between victims and aggressors. São Paulo, SP, Brazil, 2013–2023.

Relationship	Yes n (%)	No n (%)	Not known n (%)
Spouse	24,216 (30.2)	51,125 (63.8)	4,807 (6.0)
Unknown	11,868 (14.8)	63,242 (78.9)	5,038 (6.3)
Former spouse	10,629 (13.3)	64,431 (80.4)	5,088 (6.3)
Friends/acquaintances	8,114 (10.1)	66,783 (83.3)	5,251 (6.6)
Others	6,148 (7.7)	68,610 (85.6)	5,390 (6.7)
Boyfriend	3,547 (4.4)	71,421 (89.1)	5,180 (6.5)
Sibling	3,435 (4.3)	71,503 (89.2)	5,210 (6.5)
Child	2,949 (3.7)	72,134 (90.0)	5,065 (6.3)
Ex-boyfriend	2,408 (3.0)	72,537 (90.5)	5,203 (6.5)
Father	2,070 (2.6)	72,983 (91.1)	5,095 (6.4)
Mother	1,801 (2.2)	73,244 (91.4)	5,103 (6.4)
Person with an institutional relationship	1,241 (1.5)	73,909 (92.2)	4,998 (6.2)
Boss	564 (0.7)	74,568 (93.0)	5,016 (6.3)
Stepfather	485 (0.6)	74,546 (93.0)	5,117 (6.4)
Police officer/law enforcement officer	282 (0.4)	74,841 (93.4)	5,025 (6.3)
Stepmother	124 (0.2)	74,914 (93.5)	5,110 (6.4)
Caregiver	117 (0.1)	75,059 (93.7)	4,972 (6.2)

Source: Sistema de Informação de Agravos de Notificação, 2013–2023 (Notifiable Diseases Information System).

Sexism was identified as a motivation in 17.2% of cases, while generational conflicts appeared in 9.0%. It was noteworthy that 27.9% of notifications did not provide information on motivation.

### Analysis of Seasonality and Trends

**Figure 3:**
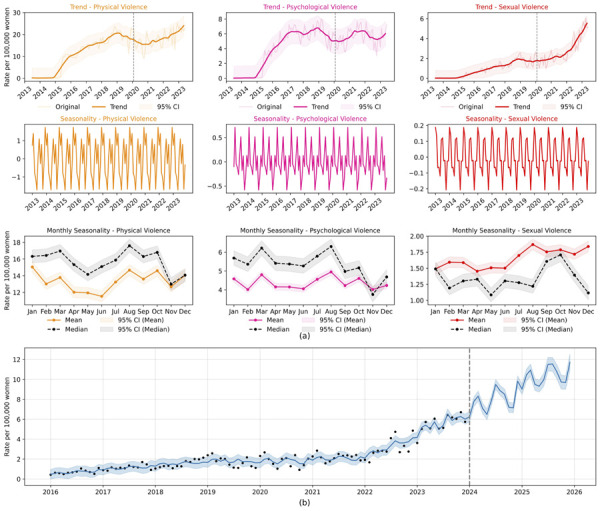
Data on violence against women in terms of seasonality and trends, São Paulo, SP, 2025.

Note: (a) Data on violence against women according to race/color (2013-2023). (b) General population data by race/color (2023). (c) Data on violence against women by type of violence (2013-2023).

Sources: Sistema de Informação de Agravos de Notificação, 2013–2023 (Notifiable Diseases Information System); Instituto Brasileiro de Geografia e Estatística (Brazilian Institute of Geography and Statistics).

This analysis is part of the “Modeling” phase of the HRSP-AI, involving decomposition of the time series and identification of seasonal patterns and trends in the records. The decomposition of the time series revealed distinct patterns for each type of violence (Figure 3A):

- Physical violence: showed an upward trend until 2019, followed by a decline and resumption of growth from 2021 onwards (post-covid-19 pandemic analysis). Seasonality showed peaks in March and August 2021, with lower levels in June.
- Psychological violence: showed moderate growth until 2019, then fell and stabilized. Similarly to physical violence, it showed seasonal peaks in March and August 2021.
- Sexual violence: showed the sharpest growth trend, especially from 2021 onwards (post-covid-19 pandemic analysis), with an increase of more than 100% compared to the pre-pandemic period. Seasonality showed a different pattern, with peaks in August and November 2021.

Predictive modeling of the rate of violence was carried out using the Prophet algorithm, with data from 2016 to 2023 and a forecast for the next 24 months, suggesting an upward trend for all types of violence and maintaining seasonal patterns. The model was configured with multiplicative seasonality (seasonality_mode=’multiplicative’), allowing the seasonal effect to vary proportionally to the magnitude of the series. Yearly seasonality (yearly_seasonality=True) was used to capture repetitive patterns throughout the year. The 95% confidence interval (interval_width=0.95) was used to estimate the uncertainty of the forecasts.

For evaluation, time series cross-validation was adopted, using the historical series from 2016 to 2022 as training and 2023 as the test period. The validation considered multiple moving windows, recalculating forecasts every six months with a 12-month horizon, comparing them with the observed data. The metrics obtained were RMSE = 1.38 and MAE = 1.14. Finally, based on the entire series from 2016 to 2023, the forecast for the next 24 months was generated, with a monthly frequency (periods=24, freq=’MS’). The code is available on [GitHub link https://github.com/andremshi/analise_violencia_mulheres for reproducibility.

### Associated Factors

Significant associations were identified between alcohol use by the aggressor and different types of violence: 1) The prevalence of sexual violence was 32% higher when the aggressor was under the influence of alcohol (PR = 1.32; 95%CI 1.25–1.40); 2) For physical violence, the prevalence was 17% higher (PR = 1.17; 95%CI 1.13–1.22); 3) Incidentally, psychological violence was 31% less prevalent in these cases (PR = 0.69; 95%CI 0.66–0.71) (Table 4).

**Table 4:** Data on violence against women regarding the statistical association between alcohol use and different types of violence. São Paulo, SP, 2025.

Violence	PR	95%CI	Observation
Sexual	1.32^a^	1.25–1.40	Prevalence of sexual violence is 32% higher among those whose perpetrator was under the influence of alcohol, compared to those whose perpetrator was not.
Physical	1.17^a^	1.13–1.22	Prevalence of physical violence is 17% higher among those whose perpetrator was under the influence of alcohol, compared to those whose perpetrator was not.
Psychological	0.69^a^	0.66–0.71	Prevalence of psychological violence is 31% lower among those whose perpetrator was under the influence of alcohol, compared to those whose perpetrator was not.

PR: prevalence ratio; 95%CI: 95% confidence interval.

^a^ For all p < 0.01.

Source: Sistema de Informação de Agravos de Notificação, 2013–2023 (Notifiable Diseases Information System).

Analysis of pregnancy status revealed that pregnant women were 2.47 times more likely to suffer sexual violence compared to non-pregnant women (PR = 2.47; 95%CI 2.46–2.48).

### Geospatial Distribution

**Figure 4:**
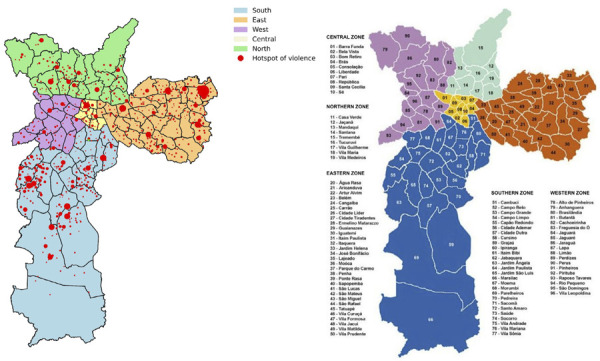
Distribution of notifications of violence against women in the districts of São Paulo, SP, Brazil. São Paulo, SP, 2025.

Source: Sistema de Informação de Agravos de Notificação, 2013–2023 (Notifiable Diseases Information System).

Also included in the “Modeling” phase, the geospatial analysis made it possible to map the records of violence according to the territorial distribution of São Paulo’s districts. The mapping of notifications by district of São Paulo revealed concentrations in peripheral areas in the south (Grajaú, Capão Redondo) and east (Itaim Paulista, Cidade Tiradentes), but also in the central region (Santa Cecília, Sé and Pari). The establishments with the highest number of notifications were Casa de Isabel (5,496), Hospital Municipal Dr. Carmino Caricchio (1,767), and Hospital Prof. Dr. Alípio Corrêa Netto (1,610) (Figure 4).

## DISCUSSION

The results showed a complex panorama of violence against women in São Paulo, demonstrating the way in which the HRSP-AI methodology made it possible to identify worrying patterns of violence. In other words, the structured application of this methodological framework identified temporal and seasonal trends in notifications, as well as characterizing specific sociodemographic vulnerabilities.

The preponderance of young, brown and black women, with less schooling and in a situation of economic dependence among the victims highlights the intersectionality of risk factors. The occupational analysis, which revealed that “housewife” was the second most frequent occupation (8.1%), behind “unemployed” (9.7%), suggests that economic vulnerability may be a factor potentially associated with violence, highlighting relationships of power and dependence that merit further research.

The predominance of young, black and brown women with less schooling among victims of violence corroborates findings in the literature. When analyzing reports of violence against women in rural contexts in Brazil between 2011 and 2020, Stochero and Pinto^21^ identified that the victims were mostly black (58.3%), with low schooling and aged between 20 and 29. Physical violence was the most common type (76.9%), followed by psychological/moral violence (41.7%) and sexual violence (8.2%). Most of the aggressors were male and intimate partners, and the aggressions took place mainly in the victims’ homes^21^. Furthermore, the 2023 Pesquisa Nacional de Violência contra a Mulher Negra (National Survey on Violence against Black Women) found that 53.0% of black victims suffered their first aggression before the age of 25, with the most common forms being psychological (87.0%), physical (78.0%), property (33.0%), and sexual (25.0%) violence^22^.

One study showed that financial dependence remains a critical factor, increasing women’s vulnerability to gender-based violence in Brazil^23^. National^6,21^ and international^29,30^ research has revealed that this dependence is one of the main reasons why many victims do not report aggression or remain in abusive relationships. Recent government initiatives, such as the creation of the Casas da Mulher Brasileira (Brazilian Women’s Houses) with programs aimed at financial independence, represent important steps, but are still insufficient given the scale of the problem shown in the domestic violence statistics^24^.

The seasonal patterns identified and the associations with alcohol use and pregnancy offer potentially impactful points of intervention for targeted public policies. The time trends observed, with a decline during the Covid-19 pandemic and a subsequent resumption of growth from 2021 onwards, suggest possible underreporting during the period of social isolation, when victims potentially found it more difficult to access health services and report aggression^2,4^.

This tendency is also supported by literature. Koutaniemi and Einiö^25^ analyzed data from Google searches and calls to the police in Finland, identifying peaks in searches for terms related to domestic violence in the months of November, January, and March, coinciding with increases in police calls categorized as domestic violence. In addition, Khurana^26^ examined data from hospital emergencies in the United States, finding that holidays such as New Year’s Eve and Independence Day are associated with domestic violence, suggesting that holidays are high-risk periods for such incidents.

Post-pandemic behavior, especially the more than 100% increase in cases of sexual violence compared to the pre-pandemic period, requires special attention in terms of public health. Predictive modeling with the Prophet algorithm reinforces this concern by suggesting a continued upward trend for all types of violence over the next 24 months, maintaining the seasonal patterns identified, which highlights the need to plan preventive and care actions considering this projected scenario.

The HRSP-AI implications, based on the adaptation to the health research context, showed its applicability as a structured methodological framework, allowing systematic approaches, from the conception to the implementation of analytical models. The main contributions of this adaptation include: 1) Structuring the research process: the Research Understanding phase provided a solid basis for defining objectives, scope and justification, guiding subsequent analyses; 2) Iterativity and flexibility: the cyclical nature of CRISP-DM favored the continuous refinement of models and approaches, essential in complex research such as violence analysis; 3) Methodological transparency: the structured documentation of each phase promotes reproducibility and peer review of the study; and 4) Integration between domain knowledge and analytical techniques: the methodology facilitated the incorporation of specific public health knowledge with advanced data analysis methods.

It is worth noting that other initiatives to adapt the CRISP-DM have been proposed in the literature^14,15,27^. The study by Areco et al.^15^ incorporated additional stages to the classic CRISP-DM, focusing on understanding the problem, planning resources, understanding and preparing the data, validation and final distribution of the dataset, making up a non-cyclical linear flow aimed at producing reliable bases for subsequent analysis. The articulation between these two models shows that the methodological structure based on CRISP-DM can be expanded according to the operational and analytical needs of each project, contributing to both the technical preparation of data and the development of predictive and strategic analyses in public health^15^.

Regarding patterns of violence and implications for public policies, the results highlighted important aspects of violence against women in São Paulo. The predominant profile of the victims (young, brown/black, with less schooling) suggests intersecting social, economic and racial vulnerabilities that should be considered in prevention and intervention policies.

The significant increase in notifications from 2015 onwards, coinciding with the implementation of Ordinance No. 1,271/2014, suggests a positive impact of this regulation in raising awareness and guiding professionals towards notification. However, the high percentage of information ignored in important fields of the records indicates a need to improve training in data collection.

The seasonality identified, with peaks in March and August in the period from 2013 to 2023, merits in-depth investigation into cultural, economic or social factors that may influence these patterns, allowing for better targeting of campaigns, educational actions, and allocation of resources.

The strong association between pregnancy and sexual violence (PR = 2.47) is an alarming finding that demands special attention, alerting us to the need for specific prenatal screening and protection protocols, considering this period of special vulnerability. This association may reflect dynamics of control and power in intimate relationships that intensify during pregnancy, pointing to the need for targeted interventions.

The increased risk of sexual violence during pregnancy found in this study (PR = 2.47) is consistent with the results of Guo et al.^28^, who, in a meta-analysis including 23 studies, identified relevant associations between intimate partner violence during pregnancy and negative outcomes such as premature birth, low birth weight and stillbirth^28^.

The identification of significant associations between alcohol use by the aggressor and the different types of violence, with an increase in the prevalence of sexual violence (32%) and physical violence (17%), but a reduction in psychological violence (31%), suggests different mechanisms of action depending on the type of violence. This difference may indicate that, under the influence of alcohol, aggressors tend to manifest more impulsive and physical behavior, while psychological violence may involve more planning or control.

The association between alcohol use and types of violence is supported by literature. International studies show that alcohol consumption by third parties, especially intimate partners, is directly related to serious forms of interpersonal violence, including physical and sexual aggression, often accompanied by psychological suffering for the victims^29,30^. This pattern is consistent with the findings of the present study, which identified a greater association between alcohol use and forms of violence that require less cognitive elaboration, such as physical and sexual violence, and a lower relationship with psychological violence.

In the context of the digital transformation of health, the structured application of methodologies such as HRSP-AI represents a significant advance for digital health, offering consistent ways of converting large volumes of data into actionable information. The systematic analysis of SINAN records demonstrates the untapped potential of existing health information systems in Brazil, which can be enhanced by advanced data science techniques. As highlighted in the literature, secondary data represents a valuable source for public health research, accentuated by its wide availability, scope and coverage^31,32^.

In this sense, the results obtained in the analysis demonstrate the practical potential of digital health. The identification of seasonal patterns, risk profiles and specific associations, such as the high risk among pregnant women and the correlation with alcohol use by the aggressor, exemplifies how digital health can transform administrative data into real-time surveillance and response tools.

The adoption of structured methodologies, such as the CRISP-DM adapted for the health context, responds directly to the WHO guidelines for the digital transformation of health systems, which recommend the use of standardized and reproducible approaches to large-scale data analysis^33^. These methods contribute to the systematization of the data life cycle, from collection and preparation to the modelling and implementation of decision-oriented digital solutions. A relevant example is the study by Cruz-Mendoza et al.^34^, who applied machine learning techniques to real databases with the aim of improving prevention, institutional response and understanding of the factors associated with violence against women^34^. This experience highlights the potential of digital approaches based on well-defined analytical processes, such as those proposed by CRISP-DM, to tackle complex public health challenges and strengthen the evidence-based intersectoral response.

In conclusion, the creation of AI models applied to public health requires methodologies that are clear, ethical and adapted to the context of the data. The HRSP-AI methodology proved to be effective in guiding this process, making it possible to identify worrying patterns in reports of violence against women in São Paulo, including increasing trends, marked seasonality and risk factors.

The use of AI in predictive modeling of time series (with the Prophet algorithm) can provide a solid basis for planning preventive actions and is a methodology that can be replicated for the analysis of other health problems.

The integration of data science and public health, structured by robust methodologies such as the one presented, represents an important advance for the development of evidence-based policies and interventions. It is recommended that this methodology be more widely adopted in public health research and that reporting systems be strengthened.

Within the scope of the HRSP-AI, the Deployment stage plays a fundamental role in guiding the conversion of analytical findings into decision support tools, technical products and inputs for public policies. The results obtained in this study – such as the identification of seasonal patterns, risk factors and territorial distribution of notifications – can support the creation of georeferenced dashboards, specific prenatal protocols, and local prevention and health surveillance strategies. This reinforces the practical applicability of the framework as an analytical structure and the basis for evidence-based interventions.

### Study Limitations

Limitations in interpreting the results: 1) Underreporting: the data represents cases reported to the health system and does not capture all occurrences. 2) Quality of records: the high percentage of ignored information in some variables limits more in-depth analysis. 3) Geographical scope: limited to the municipality of São Paulo. 4) Causality: the associations identified do not necessarily imply causal relationships, requiring further studies.

## Data Availability

Data is available on request from the corresponding author.
