# Supplementary material for: Using artificial intelligence to analyze violence against women in São Paulo
Source: Rev Saude Publica. 2026 Jul 24;60:e28. doi: 10.11606/s1518-8787.2026060007099 (PMC13398609; doi:10.11606/s1518-8787.2026060007099)
Supplement: Translation

# Translation: Uso de inteligência artificial para análise da violência contra mulheres em São Paulo

**Authors:** Paulo Bandiera-Paiva, Andre Massahiro Shimaoka, Antonio Carlos da Silva, Leonardo Martins Araujo, Débora Dupas Gonçalves do Nascimento, Maria Elisabete Salvador, Marcelo Batista Ribeiro, Kelsy Catherina Nema Areco, José Marcio Duarte, Nacime Salomão Mansur, Dulce Aparecida Barbosa

**Affiliations:** I Universidade Federal de São Paulo. Escola Paulista de Medicina. Departamento de Informática em Saúde. São Paulo, SP, Brasil; II Associação Paulista para o Desenvolvimento da Medicina. Hospital São Paulo. São Paulo, SP, Brasil; III Fundação Oswaldo Cruz. Campo Grande, MS, Brasil; IV Universidade Federal de São Paulo. Escola Paulista de Enfermagem. São Paulo, SP, Brasil

**Keywords:** Violência Contra as Mulheres, Mineração de Dados, Inteligência Artificial, Ciência de Dados

## Abstract

**OBJETIVO:**

Propor o *framework Health Research Standard Process for Artificial Intelligence*, adaptado da abordagem *Cross-Industry Standard Process for Data Mining* e aplicá-lo por meio de técnicas de séries temporais e inteligência artificial na análise dos dados do Sistema de Informação de Agravos de Notificação sobre violência contra mulheres em São Paulo, explorando dimensões sociodemográficas, espaciais e preditivas.

**MÉTODOS:**

A metodologia foi adaptada às pesquisas em saúde, substituindo a etapa original do *Business Understanding por Research Understanding* ao incorporar elementos técnico-científicos essenciais. Foram analisadas 80.148 notificações de violência contra mulheres entre 20 e 59 anos, residentes no município de São Paulo, no período de 2013 a 2023. As análises incluíram estatísticas descritivas, cálculo de razões de prevalência, decomposição e modelagem preditiva de séries temporais capturando tendências e sazonalidade, e análise geoespacial das notificações.

**RESULTADOS:**

Observou-se padrões temporais e características sociodemográficas das vítimas, com tendências crescentes nas notificações de violência física, psicológica e sexual após 2015 e comportamento sazonal. Entre as associações, identificou-se aumento de 32% na prevalência de violência sexual quando o agressor estava sob efeito de álcool e risco 2,47 vezes maior de violência sexual entre gestantes. A distribuição geoespacial revelou concentrações de notificações em áreas periféricas do município, como a zona sul, leste e central. A modelagem preditiva indicou que a tendência de aumento persistirá nos próximos 24 meses, com estimativas de taxas alcançando até 12 casos por 100.000 habitantes.

**CONCLUSÃO:**

A aplicabilidade do *Health Research Standard Process for Artificial Intelligence* foi eficaz como modelo para análises de dados e desenvolvimento de algoritmos preditivos em saúde pública. Foi possível propor uma matriz metodológica replicável para futuras pesquisas e intervenções baseadas em evidências.

## INTRODUÇÃO

A violência contra mulheres representa um grave problema de saúde pública e direitos humanos no Brasil, refletindo desigualdades sociais, econômicas e políticas que ampliam a vulnerabilidade feminina^1^. Esse fenômeno multifacetado demanda abordagens interdisciplinares para sua compreensão e enfrentamento, incluindo o uso de tecnologias avançadas, como análise de dados por inteligência artificial (IA) que oriente ações e políticas públicas.

Segundo a Organização Mundial da Saúde (OMS, 2023), aproximadamente 30% das mulheres em todo o mundo já sofreram violência física ou sexual por parceiro íntimo ao menos uma vez na vida^1^.

No Brasil, o Anuário Brasileiro de Segurança Pública (2023) revelou um cenário alarmante, com o registro de 1.437 feminicídios em 2022, representando um aumento de 6,1% em relação ao ano anterior^2^. Já a pesquisa do Instituto DataSenado de 2023 mostrou que 31% das mulheres entrevistadas no estado de São Paulo relataram ter sofrido algum tipo de violência doméstica ou familiar^3^.

Durante a pandemia de covid-19, observou-se intensificação deste cenário, com aumento de 22% nas denúncias pelo Disque 180 entre março e dezembro de 2020, evidenciando que situações de crise tendem a agravar a vulnerabilidade feminina^2,4^.

Nessa perspectiva, o Sistema de Informação de Agravos de Notificação (Sinan) constitui uma fonte valiosa de dados epidemiológicos no Brasil, ao registrar casos de violência doméstica, sexual e outras formas de agressão^5,6^. O Sinan é alimentado, sobretudo, pela notificação e investigação de casos de doenças e agravos que constam da lista nacional de doenças de notificação compulsória (Portaria de Consolidação nº 4, de 28 de setembro de 2017).

De fato, o Sinan constitui um instrumento importante para o planejamento da saúde, definição de prioridades e avaliação do impacto de intervenções. Contudo, a complexidade e o volume desses dados apresentam desafios importantes para sua análise e para a transformação dessas informações em conhecimento acionável.

O uso de ciência de dados e IA ainda é incipiente no contexto do Sistema Único de Saúde (SUS), embora algumas iniciativas já estejam em andamento, como InfoGripe e InfoDengue, que utilizam dados do Sinan para monitoramento epidemiológico^7,8^, além de ferramentas como EAI Pelotas^9^ e o Laura *Digital Emergency Room* ^10^, testadas de forma isolada em algumas unidades do SUS para apoiar a atenção hospitalar e a gestão da atenção primária à saúde. Contudo, essas ações ainda ocorrem de forma pontual e frequentemente carecem de metodologias estruturadas que orientem de maneira sistemática todas as etapas de construção, validação e aplicação dos modelos analíticos.

Assim, este estudo tem por objetivo propor o *framework Health Research Standard Process for Artificial Intelligence* (HRSP-AI) como uma estrutura metodológica e replicável para a criação de modelos analíticos e preditivos em saúde pública; aplicar essa estrutura à análise de dados do Sinan sobre violência contra mulheres em São Paulo, explorando diferentes dimensões analíticas como sociodemográfica, espacial, temporal e preditiva.

## MÉTODOS

### Desenho Local e Período do Estudo

Trata-se de um estudo metodológico, de abordagem quantitativa, baseado em análise de dados secundários, realizado no Departamento de Informática em Saúde da Escola Paulista de Medicina da Universidade Federal de São Paulo em parceria com a Associação Paulista Para o Desenvolvimento da Medicina, no período entre novembro de 2024 a maio de 2025.

Todos os dados utilizados são públicos, anonimizados e foram tratados de acordo com os princípios éticos e regulatórios vigentes, conforme a Lei Geral de Proteção de Dados Pessoais (Lei nº 13.709/2018) e a Resolução nº 510/2016 do Conselho Nacional de Saúde. O estudo foi aprovado pelo Comitê de Ética em Pesquisa da Universidade Federal de São Paulo, sob o parecer nº 00263/2023.

### Fonte de Dados e População do Estudo

Utilizou-se dados do Sinan referentes a notificações de violência contra mulheres na faixa etária de 20 a 59 anos, residentes no município de São Paulo, entre 2013 e 2023. A extração evidenciou casos de agressão por meios não especificados (CID Y09), excluindo registros de lesão autoprovocada, resultando em 80.148 notificações analisadas.

Fontes complementares incluíram: dados do Ministério da Saúde e Instituto Brasileiro de Geografia e Estatística (IBGE) (dados populacionais de mulheres de 20 a 59 anos em São Paulo, SP); informações do Departamento de Informática do SUS (CID e descrições); Classificação Brasileira de Ocupações; Cadastro Nacional de Estabelecimentos de Saúde, endereço dos estabelecimentos; dados geográficos do Sistema Estadual de Análise de Dados e Prefeitura de São Paulo (mapa de São Paulo, regiões e distritos e, latitude e longitude do estabelecimento).

### Protocolo do Estudo

A partir do Sinan e das outras fontes citadas, foi construído um banco de dados com as variáveis de interesse, abrangendo as etapas descritas nesta sessão do artigo.

Diante das adversidades metodológicas na análise de grandes volumes de dados em saúde pública, recorreu-se às metodologias estruturadas de mineração de dados, como o *Cross-Industry Standard Process for Data Mining* (CRISP-DM)^11^, reconhecido por oferecer uma abordagem sistemática para a extração de conhecimento a partir de dados massivos^12,13^. Embora eficaz em contextos corporativos, o CRISP-DM foi originalmente concebido para o setor empresarial e não contempla aspectos fundamentais da pesquisa em saúde, como o planejamento científico e os cuidados éticos no uso de dados^14,15^. No entanto, sua estrutura pode ser adaptada para incorporar esses elementos, tornando-se um roteiro metodológico robusto para o desenvolvimento de modelos de IA aplicados à saúde pública^16,17^.

### Adaptação do CRISP-DM para Pesquisas em Saúde

O CRISP-DM tradicional é composto por seis fases: *Business Understanding, Data Understanding, Data Preparation, Modeling, Evaluation e Deployment* ^7^. Para adequação ao contexto de pesquisa em saúde, adaptou-se a primeira fase para *Research Understanding*, mantendo a estrutura cíclica e iterativa do processo original (Figura 1):

**Figura 1:** Representação gráfica do *Health Research Standard Process for Artificial Intelligence* (HRSP-AI).

*Research understanding*: compreensão da pesquisa; *Data understanding*: compreensão de dados; *Data preparation*: preparação de dados; *Modeling*: modelagem; *Evaluation*: avaliação; *Deployment*: implantação; Data: dados.

Fonte: Adaptado de Chapman et al.^7^, 2020.

1. *Research Understanding*: (adaptado do original “Business Understanding”): define objetivos e perguntas de pesquisa, elaboração da justificativa, revisão de literatura, definição do escopo (população-alvo, período do estudo, limitações e abrangência), planejamento de recursos, medidas de segurança dos dados, adequação ética e definição metodológica^18^.
2. *Data Understanding*: envolve a coleta e exploração inicial dos dados, identificação de variáveis relevantes, análise de qualidade e consistência, e compreensão preliminar dos padrões e relações.
3. *Data Preparation*: compreende a limpeza, transformação e estruturação dos dados para análise, incluindo tratamento de dados faltantes, normalização, categorização e criação de novas variáveis derivadas.
4. *Modeling*: abrange a seleção e aplicação de técnicas analíticas e algoritmos, tanto para análise descritiva quanto preditiva, incluindo decomposição de séries temporais e modelagem estatística.
5. *Evaluation*: avalia os resultados conforme os objetivos da pesquisa, utilizando métricas apropriadas e validação por especialistas em saúde.
6. *Deployment*: refere-se à implementação dos conhecimentos gerados a partir da análise, contemplando sua tradução em produtos aplicáveis, como relatórios técnicos, publicações científicas, *dashboards* interativos ou sistemas de apoio à decisão. No contexto do HRSP-AI, essa etapa visa à disseminação estruturada das evidências produzidas e ao seu uso prático na formulação de políticas e intervenções em saúde pública.

### Análise de Dados

Foram realizadas análises exploratórias descritivas, contemplando distribuições de frequências absolutas e relativas das variáveis sociodemográficas e características da violência. Calculou-se razões de prevalência (RP) com intervalos de confiança de 95% (IC95%) para investigar associações entre fatores de risco e diferentes tipos de violência.

Para a análise temporal, aplicou-se a decomposição de séries temporais por meio do método ETS (*Error, Trend, Seasonality*), a fim de identificar tendências, padrões sazonais e componentes residuais^19^. Já as análises geoespaciais foram conduzidas para mapear a distribuição das notificações por distritos administrativos do município de São Paulo, com base em dados geográficos oficiais. Todos os processamentos estatísticos foram executados utilizando-se o *Python*, com os pacotes *Scipy, Statsmodels, Shapely, Geopandas, Sklearn* e *Prophet*, para análises temporais e geoespaciais.

*Análise exploratória:* realizou-se análises de frequência e distribuições das variáveis sociodemográficas (idade, raça/cor, escolaridade e ocupação), tipificação da violência, relação com o agressor e fatores associados. Calculou-se as RP para analisar associações entre as variáveis, como o uso de álcool pelo agressor e os diferentes tipos de violência.

*Preparação de dados:* antes da aplicação das técnicas de decomposição e previsão, os dados foram preparados por meio de um *pipeline* que incluiu a formatação das datas, agregação mensal das notificações, tratamento de dados faltantes e integração com dados populacionais para o cálculo das taxas por 100.000 mulheres. Os dados categóricos faltantes foram padronizados e substituídos por “Não informado”.

*Análise temporal:* aplicaram-se técnicas de decomposição de séries temporais ETS para identificar tendências e padrões sazonais das taxas de notificações por 100.000 mulheres. A decomposição separou os componentes de: 1) Tendência: direção geral da série ao longo do tempo; 2) Sazonalidade: padrões cíclicos dentro do período; 3) Resíduo: variações aleatórias não explicadas pelos outros componentes.

*Preparação de dados para modelagem preditiva*: os dados de notificações de violência foram preparados por meio de um *pipeline* que incluiu formatação de datas, agregação mensal das notificações, verificação de dados faltantes, integração com dados populacionais e cálculo das taxas por 100.000 mulheres.

*Modelagem preditiva*: utilizou-se o algoritmo *Prophet*, baseado em técnicas de IA, para a previsão das séries temporais, aproveitando sua capacidade de capturar tendências não lineares e múltiplos padrões sazonais^20^. *Prophet* foi escolhido para modelagem e previsão por suas vantagens sobre métodos tradicionais (ARIMA e *Holt-Winters*), incluindo modelagem flexível de sazonalidades múltiplas, captura de tendências não lineares e robustez a *outliers* ^20^. As métricas de acurácia do modelo *Prophet* foram avaliadas por meio do erro médio absoluto (MAE) e da raiz do erro quadrático médio (RMSE) e da validação temporal (*time series cross-validation*) com múltiplas janelas móveis, conforme prática usual na modelagem de séries temporais em saúde pública.

*Análise geoespacial*: mapeou-se a distribuição geográfica das notificações por distritos no município de São Paulo, identificando concentrações e associando com estabelecimentos de saúde que realizaram as notificações.

## RESULTADOS

A análise dos 80.148 registros de violência contra mulheres entre 20 e 59 anos no referido município (2013–2023) está apresentada em três eixos principais, alinhados às etapas do *framework* HRSP-AI: (1) análise descritiva sociodemográfica e de características da violência (etapa “*Data Understanding*”); (2) análise temporal e preditiva (etapas “*Modeling*” e “*Evaluation*”); e (3) análise geoespacial (parte da “*Modeling*”, com foco em padrões territoriais de risco).

### Caracterização das Notificações

Nesta etapa do *framework* HRSP-AI, correspondente ao “*Data Understanding*”, foi realizada uma análise descritiva das variáveis sociodemográficas e das características dos episódios de violência notificados (Tabela 1). A distribuição etária revelou maior concentração nas faixas jovens, com maior frequência entre 20–29 anos (39,6%), especialmente nos casos de violência física, psicológica e sexual, decrescendo progressivamente nas faixas etárias superiores. A idade média das mulheres foi de 34 anos (DP = 10,02; IC95%: 33,98–34,12).

**Tabela 1:** Características sociodemográficas das vítimas de violência contra mulheres. São Paulo, SP, Brasil, 2025.

Variável	n	%
Faixa etária		
20–29	31.741	39,6
30–39	25.453	31,8
40–49	15.587	19,4
50–59	7.367	9,2
Raça/cor		
Parda	32.337	40,5
Branca	32.102	40,2
Preta	9.936	12,4
Ignorado	4.566	5,7
Amarela	671	0,8
Indígena	331	0,4
Não informado	205	0,3
Escolaridade		
Sem escolaridade	2.934	3,7
Fundamental I	8.633	10,8
Fundamental II	13.142	16,4
Ensino Médio	26.444	33,0
Ensino Superior	5.442	6,8
Ignorado	22.729	28,3
Não informado	824	1,0
Ocupações (10 principais)		
Desempregada	7.764	9,7
Dona de casa	6.465	8,1
Ignorado	6.234	7,8
Diarista	990	1,2
Estudante	966	1,2
Faxineira	855	1,1
Vendedora	682	0,9
Enfermeira	636	0,8
Auxiliar de Enfermagem	631	0,8
Operadora de caixa	524	0,7

Fonte: Sistema de Informação de Agravos de Notificação (2013–2023).

Quanto à raça/cor, observou-se uma sobrerrepresentação de mulheres pardas (40,5%) e pretas (12,4%) entre as vítimas, considerando a distribuição populacional da cidade (Figura 2A). Comparando-se com dados do IBGE (2022), nota-se uma disparidade expressiva, uma vez que a população geral de São Paulo tem aproximadamente 32,6% de pessoas pardas e pretas (Figura 2B).

**Figura 2:** Dados de violência contra mulher segunda raça/cor e tipo de violência, São Paulo, SP, 2025.

(a) Dados de violência contra mulheres quanto à sazonalidade e tendências (2013–2023). (b) Previsão de tendência e sazonalidade de violência contra mulheres até 2026.

Fonte: Sistema de Informação de Agravos de Notificação (2013–2023).

Em relação à escolaridade, 33,0% das vítimas possuíam ensino médio completo, enquanto 6,8% tinham ensino superior. Destaca-se o alto percentual de registros com escolaridade ignorada ou não informada (29,3%). A análise ocupacional revelou a categoria “dona de casa” como a segunda mais frequente (8,1%), atrás apenas de “desempregada” (9,7%).

### Características da Violência

A violência física foi predominante, presente em 79,8% dos casos, seguida pela violência psicológica (26,1%) e sexual (9,8%) (Figura 2C). Em relação aos meios de agressão, a força corporal/espancamento foi o mais comum (73,8%), seguido por ameaças (18,3%) (Tabela 2).

**Tabela 2:** Características das violências notificadas contra mulheres. São Paulo, SP, Brasil, 2013–2023.

Variável	Sim	Não	Ignorado
	n (%)	n (%)	n (%)
Tipo de violência			
Física	63.978 (79,8)	15.855 (19,8)	315 (0,4)
Psicológica	20.945 (26,1)	57.863 (72,2)	977 (1,7)
Sexual	7.895 (9,8)	70.757 (88,3)	1.496 (1,9)
Tortura	1.344 (1,7)	77.085 (96,2)	1.719 (2,1)
Negligência	934 (1,2)	77.528 (96,7)	1.686 (2,1)
Financeira	1.054 (1,3)	77.375 (96,5)	1.719 (2,2)
Outros	556 (0,7)	77.255 (96,4)	2.337 (2,9)
Meio de agressão			
Força corporal	59.122 (73,8)	19.378 (24,2)	1.648 (2,1)
Ameaça	14.640 (18,3)	62.224 (77,6)	3.284 (4,1)
Objeto cortante	4.983 (6,2)	71.733 (89,5)	3.432 (4,3)
Objeto contundente	4.348 (5,4)	72.277 (90,2)	3.523 (4,4)
Outros	4.317 (5,4)	71.117 (90,2)	4.714 (4,4)
Enforcamento	4.096 (5,1)	72.633 (90,6)	3.416 (4,3)
Arma de Fogo	920 (1,1)	75.678 (94,4)	3.550 (4,4)
Objeto quente	417 (0,5)	76.205 (95,1)	3.526 (4,4)
Envenenamento	467 (0,6)	76.079 (94,9)	3.602 (4,5)
Sexo dos agressores			
Masculino	58.221 (72,6)	-	-
Feminino	11.527 (14,4)	-	-
Ambos os sexos	6.935 (8,7)	-	-
Ignorado	3.465 (4,3)	-	-
Motivação			
Ignorado	22.361 (27,9)	-	-
Outros	20.465 (25,6)	-	-
Não se aplica	13.814 (17,3)	-	-
Sexismo	13.778 (17,2)	-	-
Conflito geracional	7.187 (9,0)	-	-
Situação de rua	1.062 (1,3)	-	-
Deficiência	608 (0,8)	-	-
Homofobia, lesbofobia, bifobia ou transfobia	510 (0,6)	-	-
Racismo	152 (0,2)	-	-
Intolerância religiosa	67 (0,1)	-	-
Xenofobia	55 (0,1)	-	-

Fonte: Sistema de Informação de Agravos de Notificação (2013–2023).

Os principais autores da violência foram indivíduos do sexo masculino (72,6%), sendo cônjuges os agressores mais frequentes (30,2%), seguidos por ex-cônjuges (13,3%) e desconhecidos (14,8%) (Tabela 3).

**Tabela 3:** Relação entre a vítima e os agressores. São Paulo, SP, Brasil, 2013–2023.

Relação	Sim n (%)	Não n (%)	Ignorado n (%)
Cônjuge	24.216 (30,2)	51.125 (63,8)	4.807 (6,0)
Desconhecido	11.868 (14,8)	63.242 (78,9)	5.038 (6,3)
Ex-cônjuge	10.629 (13,3)	64.431 (80,4)	5.088 (6,3)
Amigos/conhecidos	8.114 (10,1)	66.783 (83,3)	5.251 (6,6)
Outros	6.148 (7,7)	68.610 (85,6)	5.390 (6,7)
Namorado(a)	3.547 (4,4)	71.421 (89,1)	5.180 (6,5)
Irmão(ã)	3.435 (4,3)	71.503 (89,2)	5.210 (6,5)
Filho(a)	2.949 (3,7)	72.134 (90,0)	5.065 (6,3)
Ex-namorado(a)	2.408 (3,0)	72.537 (90,5)	5.203 (6,5)
Pai	2.070 (2,6)	72.983 (91,1)	5.095 (6,4)
Mãe	1.801 (2,2)	73.244 (91,4)	5.103 (6,4)
Pessoa com relação institucional	1.241 (1,5)	73.909 (92,2)	4.998 (6,2)
Chefe/patrão	564 (0,7)	74.568 (93,0)	5.016 (6,3)
Padrasto	485 (0,6)	74.546 (93,0)	5.117 (6,4)
Policial/agente da lei	282 (0,4)	74.841 (93,4)	5.025 (6,3)
Madrasta	124 (0,2)	74.914 (93,5)	5.110 (6,4)
Cuidador	117 (0,1)	75.059 (93,7)	4.972 (6,2)

Fonte: Sistema de Informação de Agravos de Notificação (2013–2023).

O sexismo foi identificado como motivação em 17,2% dos casos, enquanto conflitos geracionais apareceram em 9,0%. Destacou-se que 27,9% das notificações não apresentavam informação sobre motivação.

### Análise da Sazonalidade e Tendências

Esta análise se insere na fase de “*Modeling*” do HRSP-AI, envolvendo a decomposição das séries temporais e identificação de padrões sazonais e tendências nos registros. A decomposição das séries temporais revelou padrões distintos para cada tipo de violência (Figura 3A):

**Figura 3:** Dados de violência contra mulheres quanto à sazonalidade e tendências, São Paulo, SP, 2025.

Nota: (a) Dados de violência contra mulheres segundo raça/cor (2013–2023). (b) Dados da população geral segundo raça/cor (2023). (c) Dados de violência contra mulheres quanto ao tipo de violência (2013–2023).

Fontes: Sistema de Informação de Agravos de Notificação (2013–2023); Instituto Brasileiro de Geografia e Estatística (2023).

- Violência física: apresentou tendência crescente até 2019, seguida de declínio e retomada de crescimento a partir de 2021 (análise pós-pandemia de covid-19). A sazonalidade mostrou picos em março e agosto de 2021, com níveis mais baixos em junho.
- Violência psicológica: exibiu crescimento moderado até 2019, queda e estabilização posterior. Similarmente à violência física, apresentou picos sazonais em março e agosto de 2021.
- Violência sexual: apresentou a tendência de crescimento mais acentuada, especialmente a partir de 2021 (análise pós-pandemia de covid-19), com aumento superior a 100% em relação ao período pré-pandemia. A sazonalidade mostrou padrão distinto, com picos em agosto e novembro de 2021.

A modelagem preditiva da taxa de violência foi realizada utilizando o algoritmo *Prophet*, com dados de 2016 a 2023 e previsão para os próximos 24 meses, sugerindo tendência crescente para todos os tipos de violência e mantendo os padrões sazonais. O modelo foi configurado com sazonalidade multiplicativa (seasonality_mode=’multiplicative’), permitindo que o efeito sazonal varie proporcionalmente à magnitude da série. A sazonalidade anual (yearly_seasonality=True) foi utilizada para capturar padrões repetitivos ao longo do ano. O intervalo de confiança de 95% (interval_width=0.95) foi utilizado para estimar a incerteza das previsões.

Para avaliação, adotou-se validação temporal (*time series cross-validation*), utilizando a série histórica de 2016 a 2022 como treino e 2023 como período de teste. A validação considerou múltiplas janelas móveis, recalculando previsões a cada seis meses com horizonte de 12 meses, comparando-as com os dados observados. As métricas obtidas foram RMSE = 1,38 e MAE = 1,14. Por fim, em seguida, com base em toda a série de 2016 a 2023, gerou-se a previsão para os próximos 24 meses, com frequência mensal (periods=24, freq=’MS’). O código está disponível em [https://github.com/andremshi/analise_violencia_mulheres] para reprodutibilidade.

### Fatores Associados

Identificaram–se associações expressivas entre o uso de álcool pelo agressor e diferentes tipos de violência: 1) A prevalência de violência sexual foi 32% maior quando o agressor estava sob efeito de álcool (RP = 1,32; IC95% 1,25–1,40); 2) Para violência física, a prevalência foi 17% maior (RP = 1,17; IC95% 1,13–1,22); 3) A propósito, a violência psicológica foi 31% menos prevalente nestes casos (RP = 0,69; IC95% 0,66–0,71) (Tabela 4).

**Tabela 4:** Dados de violência contra mulheres quanto à associação estatística entre uso de álcool e diferentes tipos de violência. São Paulo, SP, 2025.

Violência	RP	IC95%	Observação
Sexual	1,32^a^	1,25–1,40	Prevalência de violência sexual é 32% maior entre aquelas cujo autor estava sob efeito de álcool, em comparação com aquelas cujo autor não estava.
Física	1,17^a^	1,13–1,22	Prevalência de violência física é 17% maior entre aquelas cujo autor estava sob efeito de álcool, em comparação com aquelas cujo autor não estava.
Psicológica	0,69^a^	0,66–0,71	Prevalência de psicológica é 31% menor entre aquelas cujo autor estava sob efeito de álcool, em comparação com aquelas cujo autor não estava.

RP: razão de prevalência; IC95%: intervalo de confiança de 95%.

^a^ Para todos p < 0.01.

Fonte: Sistema de Informação de Agravos de Notificação (2013–2023).

Já a análise da condição de gestação revelou que as grávidas têm 2,47 vezes mais chances de sofrer violência sexual comparadas as não gestantes (RP = 2,47; IC95% 2,46–2,48).

### Distribuição Geoespacial

Também inserida na fase de “*Modeling*”, a análise geoespacial permitiu mapear os registros de violência segundo a distribuição territorial dos distritos de São Paulo. O mapeamento das notificações por distritos de São Paulo revelou concentrações em áreas periféricas na zona sul (Grajaú, Capão Redondo) e leste (Itaim Paulista, Cidade Tiradentes), mas também na região central (Santa Cecília, Sé e Pari). Os estabelecimentos com maior número de notificações foram a Casa de Isabel (5.496), Hospital Municipal Dr. Carmino Caricchio (1.767) e Hospital Prof. Dr. Alípio Corrêa Netto (1.610) (Figura 4).

**Figura 4:** Distribuição das notificações de violência contra a mulher nos distritos de São Paulo, SP, Brasil. São Paulo, SP, 2025.

Fonte: Sistema de Informação de Agravos de Notificação (2013–2023).

## DISCUSSÃO

Os resultados mostraram um panorama complexo da violência contra mulheres em São Paulo, demonstrando a forma pela qual a metodologia HRSP-AI permitiu identificar padrões preocupantes de violência. Isto é, a aplicação estruturada deste *framework* metodológico identificou tendências temporais e sazonais nas notificações, além de caracterizar vulnerabilidades sociodemográficas específicas.

A preponderância de mulheres jovens, pardas e pretas, com menor escolaridade e em situação de dependência econômica entre as vítimas ressalta a interseccionalidade de fatores de risco. A análise ocupacional, que revelou “dona de casa” como a segunda ocupação mais frequente (8,1%), atrás apenas de “desempregada” (9,7%), sugere que a vulnerabilidade econômica pode ser um fator potencialmente associado à violência, evidenciando relações de poder e dependência que merecem aprofundamento em pesquisas futuras.

Já a predominância de mulheres jovens, negras e pardas, com menor escolaridade entre as vítimas de violência corrobora achados da literatura. Ao analisar as notificações de violência contra mulheres em contextos rurais no Brasil entre 2011 e 2020, Stochero e Pinto^21^ identificaram que as vítimas eram majoritariamente negras (58,3%), com baixa escolaridade e idade entre 20 e 29 anos. A violência física foi o tipo mais frequente (76,9%), seguida pela psicológica/moral (41,7%) e sexual (8,2%). Os agressores, em sua maioria, eram do sexo masculino e parceiros íntimos, e as agressões ocorreram sobretudo nas residências das vítimas^21^. Além disso, a Pesquisa Nacional de Violência contra a Mulher Negra de 2023 apontou que 53,0% das vítimas negras sofreram a primeira agressão antes dos 25 anos, sendo as formas mais comuns a violência psicológica (87,0%), física (78,0%), patrimonial (33,0%) e sexual (25,0%)^22^.

Autores de um estudo mostraram que a dependência financeira permanece como fator crítico, aumentando a vulnerabilidade das mulheres à violência de gênero no Brasil^23^. Pesquisas nacionais^6,21^e internacionais^29,30^revelaram que esta dependência é uma das principais razões pelas quais muitas vítimas não denunciam agressões ou permanecem em relacionamentos abusivos. Iniciativas governamentais recentes, como a criação das Casas da Mulher Brasileira com programas voltados à independência financeira, representam passos importantes, mas ainda insuficientes diante da dimensão do problema evidenciada nas estatísticas de violência doméstica^24^.

Os padrões sazonais identificados e as associações com uso de álcool e gestação oferecem pontos de intervenção potencialmente impactantes para políticas públicas direcionadas. As tendências temporais observadas, com declínio durante a pandemia de covid-19 e posterior retomada do crescimento a partir de 2021, sugerem possível subnotificação durante o período de isolamento social, quando as vítimas potencialmente encontravam maior dificuldade para acessar serviços de saúde e denunciar agressões^2,4^.

Tal propensão também encontra respaldo na literatura. Koutaniemi e Einiö^25^ analisaram dados de buscas no Google e chamadas para a polícia na Finlândia, identificando picos nas buscas por termos relacionados à violência doméstica nos meses de novembro, janeiro e março, coincidindo com aumentos nas chamadas policiais categorizadas como violência doméstica. Além disso, Khurana^26^ examinou dados de emergências hospitalares nos Estados Unidos, encontrando que datas comemorativas como o Réveillon e o Dia da Independência estão associadas à violência doméstica, sugerindo que os feriados são períodos de risco elevado para tais incidentes.

O comportamento pós-pandêmico, especialmente o aumento superior a 100% nos casos de violência sexual em relação ao período pré-pandemia, requer atenção especial em termos de saúde pública. A modelagem preditiva com algoritmo *Prophet* reforça tal preocupação ao sugerir continuidade da tendência crescente para todos os tipos de violência nos próximos 24 meses, mantendo os padrões sazonais identificados, o que destaca para a necessidade de planejamento de ações preventivas e de acolhimento considerando este cenário projetado.

As implicações HRSP-AI, a partir da adaptação ao contexto de pesquisa em saúde, mostrou sua aplicabilidade como *framework* metodológico estruturado, permitindo abordagens sistemáticas, desde a concepção até a implementação de modelos analíticos. As principais contribuições desta adaptação incluem: 1) Estruturação do processo de pesquisa: a fase de *Research Understanding* proporcionou base sólida para definição de objetivos, escopo e justificativa, direcionando as análises subsequentes; 2) Iteratividade e flexibilidade: a natureza cíclica do CRISP-DM favoreceu o refinamento contínuo dos modelos e abordagens, essencial em pesquisas complexas como análise de violência; 3) Transparência metodológica: a documentação estruturada de cada fase promove a reprodutibilidade e avaliação do estudo por pares; e 4) Integração entre conhecimento do domínio e técnicas analíticas: a metodologia facilitou a incorporação de conhecimentos específicos de saúde pública com métodos avançados de análise de dados.

Destaca-se que outras iniciativas de adaptação do CRISP-DM têm sido propostas na literatura^14,15,27^. O estudo de Areco et al.^15^ incorporou etapas adicionais ao CRISP-DM clássico, com foco no entendimento do problema, planejamento de recursos, entendimento e preparação dos dados, validação e distribuição final do *dataset*, compondo um fluxo linear não cíclico voltado à produção de bases confiáveis para análises subsequentes. A articulação entre esses dois modelos evidencia que a estrutura metodológica baseada no CRISP-DM pode ser ampliada conforme as necessidades operacionais e analíticas de cada projeto, contribuindo tanto para o preparo técnico dos dados quanto para o desenvolvimento de análises preditivas e estratégicas em saúde pública^15^.

Referente aos padrões de violência e implicações para políticas públicas, os resultados evidenciaram aspectos importantes da violência contra mulheres em São Paulo. O perfil predominante das vítimas (jovens, pardas/pretas, com menor escolaridade) sugere interseccionalidades de vulnerabilidades sociais, econômicas e raciais que devem ser consideradas em políticas de prevenção e intervenção.

O aumento expressivo nas notificações a partir de 2015, coincidindo com a implementação da Portaria nº 1.271/2014, sugere impacto positivo desta normativa na sensibilização e orientação dos profissionais para a notificação. Contudo, o alto percentual de informações ignoradas em campos importantes dos registros indica necessidade de aprimoramento na capacitação para coleta de dados.

A sazonalidade identificada, com picos em março e agosto no período de 2013 até 2023, merece investigação aprofundada sobre fatores culturais, econômicos ou sociais que possam influenciar estes padrões, permitindo melhor direcionamento de campanhas, ações educativas e alocação de recursos.

A forte associação entre gestação e violência sexual (RP = 2,47) constitui um achado alarmante que demanda atenção especial, alertando para a necessidade de protocolos específicos de triagem e proteção no pré-natal, considerando este período de especial vulnerabilidade. Esta associação pode refletir dinâmicas de controle e poder nas relações íntimas que se intensificam durante a gestação, apontando para a necessidade de intervenções direcionadas.

O risco aumentado de violência sexual durante a gestação encontrado neste estudo (RP = 2,47) é consistente com resultados de Guo et al.^28^, que, em meta-análise incluindo 23 estudos, identificaram associações relevantes entre violência por parceiro íntimo durante a gravidez e desfechos negativos como parto prematuro, baixo peso ao nascer e natimortalidade^28^.

A identificação de associações importantes entre o uso de álcool pelo agressor e os diferentes tipos de violência, com aumento na prevalência de violência sexual (32%) e física (17%), mas redução na violência psicológica (31%), sugere mecanismos distintos de ação conforme o tipo de violência. Esta diferença pode indicar que, sob efeito de álcool, agressores tendem a manifestar comportamentos mais impulsivos e físicos, enquanto a violência psicológica pode envolver maior planejamento ou controle.

A associação do uso de álcool com os tipos de violência encontra respaldo na literatura. Estudos internacionais mostram que o consumo de álcool por terceiros, especialmente parceiros íntimos, está diretamente relacionado a formas graves de violência interpessoal, incluindo agressões físicas e sexuais, muitas vezes acompanhadas de sofrimento psicológico para as vítimas^29,30^. Esse padrão é consistente com os achados do presente estudo, que identificou maior associação do uso de álcool com formas de violência que exigem menor elaboração cognitiva, como a violência física e sexual, e menor relação com a violência psicológica.

Referente ao contexto da transformação digital da saúde, a aplicação estruturada de metodologias como o HRSP-AI representa um avanço expressivo para a saúde digital, oferecendo caminhos consistentes para converter grandes volumes de dados em informações acionáveis. A análise sistemática de registros do Sinan demonstra o potencial inexplorado dos sistemas de informação em saúde já existentes no Brasil, que podem ser potencializados por técnicas avançadas de ciência de dados. Como destacado na literatura, os dados secundários representam uma fonte valiosa para a pesquisa em saúde coletiva, acentuados pela ampla disponibilidade, abrangência e cobertura^31,32^.

Nesse sentido, os resultados obtidos na análise demonstram o potencial prático da saúde digital. A identificação de padrões sazonais, perfis de risco e associações específicas, como o risco elevado entre gestantes e a correlação com uso de álcool pelo agressor, exemplifica como a saúde digital pode transformar dados administrativos em ferramentas de vigilância e resposta em tempo real.

Já a adoção de metodologias estruturadas, como o CRISP-DM adaptado para o contexto da saúde, responde diretamente às diretrizes da OMS e para a transformação digital dos sistemas de saúde, que recomendam o uso de abordagens padronizadas e reprodutíveis na análise de dados em larga escala^33^. Tais métodos contribuem para a sistematização do ciclo de vida dos dados, desde a coleta e preparação até a modelagem e implementação de soluções digitais orientadas à decisão. Exemplo relevante é o estudo de Cruz-Mendoza et al.^34^, que aplicaram técnicas de aprendizado de máquina sobre bases de dados reais com o objetivo de aprimorar a prevenção, a resposta institucional e a compreensão dos fatores associados à violência contra mulheres^34^. Essa experiência evidencia o potencial de abordagens digitais fundamentadas em processos analíticos bem definidos, como os propostos pelo CRISP-DM, para enfrentar desafios complexos da saúde pública e fortalecer a resposta intersetorial baseada em evidências.

Em conclusão, a criação de modelos de IA aplicados à saúde pública exige metodologias claras, éticas e adaptadas ao contexto dos dados. A metodologia HRSP-AI mostrou-se eficaz para orientar esse processo, o que possibilitou a identificação de padrões preocupantes nas notificações de violência contra mulheres em São Paulo, incluindo tendências crescentes, sazonalidade marcada e fatores de risco.

O uso de IA na modelagem preditiva de séries temporais (com o algoritmo *Prophet*) pode fornecer uma base sólida para o planejamento de ações preventivas, sendo uma metodologia passível de replicação para a análise de outros agravos à saúde.

A integração entre ciência de dados e saúde pública, estruturada por metodologias robustas como a apresentada, representa um avanço importante para o desenvolvimento de políticas e intervenções baseadas em evidências. Recomenda-se a adoção mais ampla dessa metodologia em pesquisas em saúde pública e o fortalecimento dos sistemas de notificação.

No âmbito do HRSP-AI, a etapa de *Deployment* assume papel fundamental ao orientar a conversão dos achados analíticos em ferramentas de suporte à decisão, produtos técnicos e insumos para políticas públicas. Os resultados obtidos neste estudo – como a identificação de padrões sazonais, fatores de risco e distribuição territorial das notificações – podem subsidiar a criação de *dashboards* georreferenciados, protocolos específicos no pré-natal, e estratégias locais de prevenção e vigilância em saúde. Assim, reforça-se a aplicabilidade prática do *framework* como estrutura analítica e base para intervenções orientadas por evidências.

### Limitações do Estudo

Limitações na interpretação dos resultados: 1) Subnotificação: os dados representam casos notificados ao sistema de saúde, não capturando a totalidade das ocorrências. 2) Qualidade dos registros: o alto percentual de informações ignoradas em algumas variáveis limita análises mais aprofundadas. 3) Escopo geográfico: limitado ao município de São Paulo. 4) Causalidade: as associações identificadas não implicam necessariamente relações causais, demandando estudos complementares.

## Data Availability

Os dados estão disponíveis mediante solicitação ao autor correspondente.
